# Technostress in Spanish University Teachers During the COVID-19 Pandemic

**DOI:** 10.3389/fpsyg.2021.617650

**Published:** 2021-02-25

**Authors:** Maria Penado Abilleira, María-Luisa Rodicio-García, María Paula Ríos-de Deus, Maria José Mosquera-González

**Affiliations:** ^1^Facultad de Ciencias de la Salud, Universidad Isabel I, Burgos, Spain; ^2^Unidad de Investigación FORVI (Formación y Orientación Para la Vida), Universidad de A Coruña, A Coruña, Spain

**Keywords:** technostress, university, teacher, job performance, COVID-19

## Abstract

One of the measures adopted by the government of Spain during the COVID-19 pandemic has been the elimination of face-to-face classes in all universities, requiring that all teachers had to conduct their classes in an online mode. The objective of this article is to study how this adaptation among university teachers affected their job performance due to the technostress (objective and subjective) that they may have suffered. Based on the person-environment misfit theory (P-E fit theory), the sample consisted of 239 teachers from face-to-face and online universities in Spain who were asked to identify the type of technostress, feelings of technostress, and impact on job performance as a result of online teaching due to the COVID-19 pandemic. Results show that teachers who suffered the most from the negative consequences of technology have been female teachers from face-to-face universities who are older, have more years of experience, and consequently, hold a higher position. Despite previous results none of the above variables have been significant in explaining the decline in job performance during confinement. It was also observed that although the effect on job performance was similar for online teachers as well as face-to-face teachers, the variables that explained this effect were different. For the online teachers, there was a misfit between the demands and resources, which are explained based on the previous theory (P-E fit theory). Teachers from face-to-face universities pointed to the lack of instructions from their organization, along with subjective feelings of techno-inefficacy, as the reasons behind the decline in job performance during the lockdown period. Looking ahead to future research on the incorporation of information and communications technology in teaching work, it is necessary to consider variables associated with technostress, both objective and subjective, in order to increase the effectiveness of integrating emerging technology into teaching work.

## Introduction

It was in the 1980s, in the book *Technostress: The Human Cost of the Computer Revolution* (Brod, 1984), that technostress was first spoken of as an adaptive disease caused by people’s inability to face new technologies in a healthy way. Since then, many authors have attempted to define the term by broadening and qualifying these initial assumptions. Some, like Weil and Rosen (1997) have spoken of negative impacts on attitudes, thoughts, or behaviors, caused directly or indirectly through technology.

In the Spanish context, Salanova (2003) refers to technostress associated with the use of information and communications technology (ICT) as follows:

A negative psychological state related to the use of ICT or a threat to its use in the future. This state is conditioned by the perception of a mismatch between demands and resources related to the use of ICTs, leading to a high level of unpleasant psychophysiological activation and the development of negative attitudes towards ICT (Salanova, 2003, p. 231).

Following this definition, Llorens et al. (2011) focused their efforts on studying the different components of the subjective experience of technostress by grouping them into what they called *technostrain*. This concept is understood as the negative psychological experience derived from the stress that occurs when using technology (Llorens et al., 2011). For the authors, this would configure the affective dimension of technostress syndrome. *Technostrain* includes anxiety (techno-anxiety) and fatigue (techno-fatigue) related to technology, skepticism (techno-skepticism) caused by it, and inefficacy (techno-inefficacy) when using technological resources.

The anxiety dimension (techno-anxiety) includes psychological anxiety (fear of damaging the computer), social anxiety (fear of being replaced by a machine), and anxiety in operation (inability to use technology). In addition to anxiety, people experience feelings of fatigue (techno-fatigue), tiredness, and mental and cognitive exhaustion (e.g., “When I finish working with technology, I feel exhausted”) due to the use of technology. This fatigue is related to the development of negative attitudes toward technology (Salanova et al., 2007).

Skepticism (techno-skepticism) constitutes the attitudinal dimension of technostress and refers to the negative evaluations generated by the use of technology, such as an indifference or a disconnected attitude toward technology (e.g., “As times goes by technology interests me less and less”) (Llorens et al., 2011).

Finally, Llorens et al. (2011) differentiate the cognitive dimension of technostress (techno-ineffectiveness) by describing it as negative thoughts about one’s ability to use technology successfully (e.g., “In my opinion, I am ineffective using technology”).

Users who experience *technostrain* will present high levels of unpleasant physiological activation that materialize in anxiety, tension, and discomfort due to the current or future use of ICT. The user may experience anxiety due to a feeling of not having enough time to respond to the amount of digital data that they receive in their day-to-day work. One example of this may be the immediacy of responding to incoming emails or mobile messages in a short time. On the other hand, users may have a negative attitude toward the use of ICTs because they may think that they are a hindrance due to errors caused by the computer system or to their work process. These users do not see the benefits of ICT because they are sometimes not capable of using them.

In addition to the subjective experience or sensation of technostress, multiple theories have tried to objectify this phenomenon, indicating that the unpleasant sensation of technostress is produced by an imbalance between people and the technological environment in which they carry out their work, focusing on objective variables instead of the subjective sensation or the feeling that they cause in the person.

Within this objective vision of the phenomenon of the technostress person-environment misfit theory (P-E fit theory) (Harrison, 1978; Edwards, 1996; Edwards et al., 1998), an assumption exists that there is an equilibrium between people and their environment; when this relationship is out of balance, tension is generated (Ayyagari et al., 2011). Stress is caused neither by the person nor the environment but appears when there is no adjustment between the two (e.g., between the needs of the person and the resources of the environment, or between the aptitudes and abilities of the person and the demands of the environment). Thus, technostress is conceptualized as a misfit between a person and the environment. It is not only limited by technology itself but also by the organization that has established the requirements for its use, and the members of the organization that, on multiple occasions, have an influence on the individual’s use of technology (Avanzi et al., 2018).

Most of the studies on the negative effects of technostress have focused on a business or industrial work context (Ragu-Nathan et al., 2008; Jung et al., 2012; Fuglseth and Sørebø, 2014; Jena, 2015; Tarafdar et al., 2015; Hsiao, 2017; Marchiori et al., 2019; Salanova, 2020); however, an increasing amount of research is currently being focused on the educational context (Özgür, 2020; Penado Abilleira et al., 2020).

Llorens et al. (2011) point out that teaching is one of the most stressful professions in the world due to continuous changes derived from scientific and technological advances that have occurred from the 1990s to the present. Today, the role of the teacher has evolved from a simple “transmitter of knowledge” to a “complex designer of learning environments” (Gros and Silva, 2005), where technology is used as a teaching-learning method. Teachers today must attend to the interaction between three main components of the learning environment: content, pedagogy, and technology. This is considered necessary as teachers have the so-called “knowledge of technological pedagogical content” (TPACK) (Koehler and Mishra, 2005; Mishra and Koehler, 2006; Rienties et al., 2013; Özgür, 2020; Schildkamp et al., 2020). Today’s teachers are expected to integrate technology both positively and effectively into their teaching in the classroom (Graham et al., 2009), and they constantly struggle with the time available to keep pace with emerging technology and with the associated innovations in pedagogy (Tarus et al., 2015; Voet and De Wever, 2017). In addition, teachers usually see technology as tools for lesson preparation, knowledge delivery, or to attract students, but they lack adequate skills and competencies in designing and implementing the constructive use of technology in the teaching and learning process (Chen, 2008; Munyengabe et al., 2017). The continuous upgrading of technology exposes teachers to constant technostress because teachers do not always have the knowledge required to use new and updated technologies (Altınay-Gazi and Altınay-Aksal, 2017; Li and Wang, 2020). However, teachers’ ability to integrate technology into classroom pedagogically is crucial to educational innovation (Koh et al., 2017; Schildkamp et al., 2020).

Based on the P-E fit theory, Al-Fudail and Mellar (2008) proposed a teacher-technology interaction model that shows how teachers experience technostress when there is a discrepancy between their characteristics (e.g., abilities and needs) and school technology support (e.g., training and technical support). The competencies and attributes of the teaching staff are essential when incorporating technology into teaching work. This statement is even more evident in times of a pandemic; hence, there is a proliferation of studies that have focused attention on this topic and have demonstrated the extent to which this research is important (Bruggeman et al., 2021).

Currently, university teaching work is developed in a context where technology is very present but is not always well integrated into daily teaching. The lockdown of teaching centers caused by the pandemic has left teachers improvising new forms of teaching resembling “emergency remote teaching” (Hodges et al., 2020; Mohmmed et al., 2020), than quality online teaching.

Of the many ways in which teaching is being carried out today, we understand that the daily work of online universities is 100% based on technology as a means of approaching students, transmitting content, and carrying out evaluation activities, without any type of in-person contact. On the other hand, face-to-face teaching uses personal contact in situations as a fundamental strategy of the teacher-learner process, regardless of the fact that there are virtual platforms that complement this action and support the content transmission process.

Understanding these two teaching modalities in these terms, it can be assumed that online universities, in principle, have implemented more ways of acting through ICT and that, therefore, their faculty members may experience less misfit (technostress) between the demands of the institution and their own needs regarding technology. In face-to-face universities, these imbalances may be greater due to the lack of a tradition of fully integrating technology in teaching.

Until now, the study of technostress has been carried out either objectively or by considering the subjective nature of the said phenomenon. Therefore, an integrative vision that relates both spheres within the work environment is necessary. Based on the above, the purpose of the present investigation is to study the levels and types of technostress that professors at a Spanish university reported during the isolation and confinement measures of the population due to the COVID-19 pandemic. During this period, all teaching activities of the university had to be conducted online.

Based on the hypothesis that, in online universities, teachers receive the necessary support to overcome technostress caused by technological inadequacy and that, in contrast, face-to-face universities have had to adapt quickly to online teaching without sufficient training and with a high level of improvisation, a higher level of technostress is expected in teachers from face-to-face universities, compared to those who routinely performed their teaching functions online.

## Materials and Methods

### Procedure and Participants

The sample comprised of 239 teachers (46.6% men and 53.4% women) from Spanish universities, aged between 26 and 69 years (*M* = 47.03; SD = 10.17). The participating teachers had experience teaching for an average of 15 years (SD = 12.20).

Among the participating university professors, 71.5% were at universities that, without taking into account the measures established during confinement, carried out 100% of their teaching through direct student-teacher interaction (i.e., face-to-face). In this scenario, students were required to travel to the university in order to receive content from teachers without using any type of digital or online platform. The remaining teachers (28.5%) only had experience interacting with their students through the use of virtual classrooms or digital platforms (i.e., without any physical contact between the student and the teacher).

The questionnaire was sent via email to participants to specify the objectives of the research, identification of the authors of the study, and anonymity of the answers provided. The personal data collected would not allow for the identification of the teachers, thus complying with the indications received by the ethics committee of the universities involved and the regulation of personal data, as well as the recommendations of the Declaration of Helsinki (2016/679) approved by the European Parliament of the European Union.

Teachers were asked for their consent to participate in the study and to extract information for the sole and exclusive purpose of the research. To do this, a mandatory question was introduced prior to viewing the questionnaire, which, if not answered in the affirmative, prevented completion of the questionnaire.

The questionnaire was distributed via email using the official distribution lists of the participating universities. The response percentage has been 10%, which is in line with participation rates in similar types of research (Li and Wang, 2020; Özgür, 2020).

Data collection began in mid-April (April 17, 2020), which coincided with the month of confinement of the population due to the state of alarm decreed by the government of Spain caused by the COVID-19 pandemic, and ended a month later (May 16, 2020), when the first deconfinement measures occurred.

During the data collection period, all face-to-face teaching activities at official education centers (e.g., nursery schools, primary, secondary, high schools, vocational training centers, and universities) were suspended and had to be carried out online or remotely.

### Measures

The **technostress questionnaire** (Wang and Li, 2019), based on a multidimensional person-environment model to estimate the phenomenon of technostress among university teachers, was used. In the instrument, technostress was conceptualized as the result of a maladjustment or misfit in three main areas of people’s interaction with the environment in which they work: from person to organization (P-O; person-organization misfit), from person to technology (P-T; person-technology misfit), and from people to each other (P-P; person-people technology). The maladjustment of people to the organization and technology was also conceptualized using a double path: on the one hand, the lack of abilities of the subjects and, on the other, a lack of resources to adapt to changes.

The misfit of the person to the organization (P-O) encompasses both the maladjustment of the abilities of the subjects in relation to the new demands of their job conditions (A-D; abilities-demands misfit) as well as the lack of support or resources on the part of the institution in the face of the new needs of teachers (N-S; needs-supplies misfit).

The misfit of the person to technology (P-T) assumes that the technological skills of the teachers will quickly become obsolete due to the constant change in the technological and information systems, which forces them to work faster and with greater technological demands (A-D; abilities-demands misfit). Likewise, the inappropriate use of technology may result from the use of technological tools that are not adequate to the task or from a lack of customization of the available tools (N-S; needs-supplies misfit).

The misfit of people with each other (P-P) is conceptualized as the lack of support on the part of other colleagues when carrying out academic tasks, which can increase the feeling of uselessness of new technologies and increase technostress.

The scale proposed by the authors comprised of 22 items that the user had to rate on a five-point Likert scale (1 = Strongly disagree, and 5 = Strongly agree). In the finalized, multidimensional P-E misfit scale of technostress (total score ranging from 22 to 110), the higher the score, the greater the level of technostress. Specifically, a score of 22 indicated the absence of technostress, scores of 23–65 corresponded to a mild level of technostress, scores of 66–87 indicated a moderate level of technostress, and scores ≥88 corresponded to a severe level of technostress (Wang and Li, 2019). The original validation of the instrument showed good reliability, with Cronbach’s Alpha coefficients ranging from 0.79 to 0.90 in the assessed dimensions.

The questionnaire offered an objective measure of technostress based on the misfit between demands and resources while ignoring the subjective impact of this imbalance on the individual.

The **Salanova questionnaire** (Salanova, 2003) was used to assess the subjective sensation of technostress. This questionnaire conceives technostress as the psychosocial damage produced by technology in three dimensions: the affective dimension (anxiety vs. fatigue), the attitudinal dimension (skeptical attitude toward technology), and the cognitive dimension (beliefs of ineffectiveness in the use of technology).

The items on these scales were answered by teachers who use ICT in their work. The questionnaire was answered using a Likert-type frequency scale ranging from 0 (not at all/never) to 6 (always/every day). Internal consistency tests corroborated the reliability of the scale with a Cronbach’s Alpha score that exceeded the cut-off point of 0.70 on all scales, with subscales ranging from 0.83 to 0.93.

In order to obtain the scores of each scale (i.e., fatigue, anxiety, skepticism, and ineffectiveness), the scores of the items were added and then divided by the number of items of the scale to achieve the result. This established the ranges reflected in the table below (see Table 1).

**TABLE 1:** Scores ranges of the Salanova (2003) technostress scale.

		Anxiety	Fatigue	Skepticism	Inefficacy
Very Low	>5%	0.00	0.00	0.00	0.00
Low	5–25%	0.01–0.25	0.01–0.25	0.00	0.00
Medium (Low)	25–50%	0.26–1.00	0.26–1.00	0.01–1.00	0.01–0.75
Medium (High)	50–75%	1.01–2.00	1.01–2.25	1.01–2.00	0.76–1.75
High	75–95%	2.01–3.25	2.26–4.18	2.01–4.01	1.76–3.02
Very High	>95%	>3.25	>4.18	>4.01	>3.02

To observe the effect of technostress (in its objective and subjective dimensions) on the performance of university teachers, a questionnaire adapted from Tarafdar et al. (2010) for the estimation **of job performance** was used. This questionnaire contained six elements. Examples of these elements include: “*ICT in my university improves the quality of my work*” and “*ICT in my university improves my labor productivity*.” The same questionnaire was used in the research by Wang and Li (2019). A reported Cronbach’s Alpha of 0.91 was obtained for this instrument.

### Statistical Analyses

To obtain the psychometric properties of the questionnaire, a reliability analysis was performed by calculating Cronbach’s Alpha statistic.

For the comparison of the scores obtained between the different groups, Student *t*-tests or Chi-square tests were performed depending on the type of variable used. The correlations observed between the scales and subscales of the instruments were obtained using Pearson’s R statistics.

To estimate the predictive models of technostress in the teaching performance of university professors, a structural equation model (SEM) was carried out with the considered variables. The estimation method was unweighted least squares (ULS), and to value the adjustment of the model, the following indices were used: goodness-of-fit index (GFI), adjusted goodness-of-fit index (AGFI), root mean square residual index (RMR), normed fit index (NFI), and relative fit index (RFI). In accordance with Kline (2016), the values showed a good model fit, as RMR ≤ 0.08, and GFI, AGFI, NFI, and RFI > 0.90.

All analyses were performed using IBM SPSS statistical software (version 25) and AMOS extension for SPSS.

## Results

### Reliability Analysis

The reliability analysis carried out on the scales and questionnaires used showed good statistical results for both the global scales and the subfactors or dimensions, exceeding the cut-off point of 0.70 (see Table 2).

**TABLE 2:** Reliability of the scales used.

	**Total**
Wang and Li, 2019	0.954
ADO	0.915
NSO	0.826
ADT	0.861
NST	0.856
PPF	0.841
Salanova, 2003	0.953
ANS	0.890
FAT	0.956
SKE	0.908
INEF	0.894
Tarafdar et al. (2010)	0.917

*ADO, abilities-demands organization misfit; NSO, needs-supplies organization misfit; ADT, abilities-demands technology misfit; NST, needs-supplies technology misfit; P-P, person-people misfit; ANS, anxiety; FAT, fatigue; SKE, skepticism; INEF, inefficacy.*

### Prevalence Analysis

The results obtained in the Wang and Li (2019) technostress questionnaire showed a mild level of technostress in the sample, with significant differences depending on the type of university in which teachers carried out their educational functions [χ^2^ (1) = 44.389, *p* < 0.001]. This indicated a greater presence of moderate technostress in the teachers from the face-to-face universities (26.3%), while 14.7% of the participants affirmed having a total absence of this variable. In relation to gender, results showed significant differences, indicating that women suffer more technostress compared to their male colleagues [χ^2^ (1) = 314.389, *p* < 0.001] (see Table 3).

**TABLE 3:** Percentage of the sample at the different levels of technostress (Wang and Li, 2019) according to the type of university and gender.

	**Total**	**Online**	**Face-to-face**	**Men**	**Women**
Absence	4.2	14.7	0	4.5	3.9
Mild	73.2	79.4	70.8	76.6	70.1
Moderate	20.1	4.4	26.3	16.2	23.6
Severe	2.5	1.5	2.9	2.7	2.4

Significant differences in the levels of technostress depending on the type of university were maintained when the average scores of each of the dimensions or factors that make up the questionnaire were considered.

Compared to their peers who performed teaching functions at an online university, teachers from face-to-face universities presented the highest scores in all the subfactors considered in the scale: those related to the organization {ADO [*t*(237) = −3.708, *p* < 0.001; *d* = 0.53]; NSO [*t*(237) = −6.694, *p* < 0.001; *d* = 0.89]}, those related to technology {ADT [*t*(237) = −5.836, *p* < 0.001; *d* = 0.87]; NST [*t*(237) = −5.435, *p* < 0.001; *d* = 0.69]}, and those related to interactions between people {PPF [*t*(237) = −6.604, *p* < 0.001; *d* = 0.91]}. According to gender, women appear to suffer greater technostress compared to men, although these differences were not considered significant (see Table 4).

**TABLE 4:** Average scores obtained in the Wang and Li (2019) technostress questionnaire according to the type of university.

	**Total**	**Online**	**Face-to-face**	**Men**	**Women**
ADO	2.1770	1.7978	2.3278	2.1667	2.1953
NSO	2.3699	1.7316	2.6238	2.4054	2.3458
ADT	2.3926	1.7721	2.6394	2.3266	2.4573
NST	2.1341	1.6382	2.3313	2.0968	2.1756
PPF	2.3421	1.6912	2.6009	2.3041	2.3819
TOTAL	2.2831	1.7262	2.5046	2.2599	2.3112

For the remaining variables, the results show a greater presence of general technostress related to age and years of teaching experience. This may indicate that the older and more experienced teachers are those who, to a greater extent, suffered the most negative consequences of technology (see Table 5).

**TABLE 5:** Pearson correlations between Wang and Li (2019) technostress questionnaire and age and years of experience.

	**Age**	**Years of experience**	**ADO**	**NSO**	**ADT**	**NNS**	**PPF**
Age	1						
Years of experience	0.672**	1					
ADO	0.285**	0.290**	1				
NSO	0.257**	0.330**	0.570**	1			
ADT	0.315**	0.362**	0.826**	0.677**	1		
NNE	0.298**	0.347**	0.702**	0.732**	0.755**	1	
PPF	0.330**	0.361**	0.549**	0.666**	0.619**	0.688**	1

**p < 0.01.

In Salanova’s (2003) technostress questionnaire, the results showed that the type of university had an influence on the subjective feeling of technostress, both on a global scale as well as in the estimated sub-dimensions.

Differences can be found by the ranges obtained in the following subscale: techno-anxiety [χ^2^ (5) = 14.706, *p* < 0.05], techno-fatigue [χ^2^ (4) = 36.034, *p* < 0.001], techno-skepticism [χ^2^ (4) = 34.983, *p* < 0.001], and techno-effectiveness [χ^2^ (4) = 21.202, *p* < 0.001]. These results indicate a greater subjective sensation of technostress in face-to-face universities than in online universities. If the gender of participants was considered, significantly higher results were observed in female teachers across all dimensions included in the questionnaire: techno-anxiety [χ^2^ (5) = 14.706, *p* < 0.05], techno-fatigue [χ^2^ (5) = 36.034, *p* < 0.001], techno-skepticism [χ^2^ (4) = 34.983, *p* < 0.001], and techno-effectiveness [χ^2^ (4) = 21.202, *p* < 0.001] (see Table 6).

**TABLE 6:** Technostress (Salanova, 2003) levels according to the type of university and gender.

	**Total**	**Online**	**Face-to-face**	**Men**	**Women**
**Techno-anxiety**					
Very low	19.7	31.3	15.2	24.5	15.7
Low	8.4	10.4	7.6	8.2	7.9
Medium low	21	22.4	20.5	28.2	15
Medium high	18.9	16.4	19.9	15.5	22
High	16.4	4.5	21.1	17.3	15.7
Very high	15.5	14.9	15.8	6.4	23.6
**Techno-fatigue**					
Very low	20.2	31.3	15.8	22.7	18.1
Low	2.9	1.5	3.5	3.6	2.4
Medium low	15.1	13.4	15.8	17.3	12.6
Medium high	19.3	23.9	17.5	20	18.9
High	21.8	19.4	22.8	20.9	22.8
Very high	20.6	10.4	24.6	15.5	25.2
**Techno-skepticism**					
Muy bajo	29.8	46.3	23.4	30	29.9
Very low		0.0	0.0	0	0
Low	18.5	20.9	17.5	16.4	19.7
Medium low	18.1	14.9	19.3	20	16.5
Medium high	26.1	14.9	30.4	28.2	24.4
High	7.6	3.0	9.4	5.5	9.4
**Techno-inefficacy**					
Very low	25.2	41.8	18.7	28.2	22.8
Low					
Medium low	23.5	29.9	21.1	24.5	22
Medium high	21.4	17.9	22.8	20	22.8
High	21.4	4.5	28.1	20.9	22
Very high	8.4		9.4	60.4	10.2

Significant differences were observed in the mean scores obtained for each of the factors considered, which, like the established ranges, point to a greater subjective feeling of technostress in teachers from face-to-face universities. Results from the following subscales include: techno-anxiety [*t*(236) = −2.749, *p* < 0.01; *d* = 0.62], techno-fatigue [*t*(236) = −3.016, *p* < 0.05; *d* = 0.82], techno-skepticism [*t*(236) = −3.984, *p* < 0.001; *d* = 0.87], and techno-inefficacy [*t*(236) = −3.799, *p* < 0.001; *d* = 0.70].

Taking into account the gender of the participants, women held higher mean scores in the Salanova questionnaire, although these differences can only be considered significant when we examined the global mean score in the instrument [*t*(235) = −2.524, *p* < 0.05; *d* = 0.38]. This resulted in the following score: techno-fatigue [*t*(235) = −2.558, *p* < 0.05; *d* = 0.63] and techno-anxiety [*t*(235) = −3.687, *p* < 0.01; *d* = 0.74] (see Table 7).

**TABLE 7:** Average scores obtained in the Salanova (2003) technostress questionnaire according to the type of university and gender.

	**Total**	**Online**	**Face-to-face**	**Men**	**Women**
Techno-anxiety	1.60	1.1604	1.7807	1.2136	1.9567
Techno-fatigue	2.24	1.6530	2.4751	1.9091	2.5433
Techno-skepticism	1.58	0.9565	1.8275	1.5159	1.6444
Techno-inefficacy	1.22	2.8756	2.7000	1.1055	1.3260
Total	1.88	1.4730	2.0399	1.6822	2.0595

As with the objective technostress questionnaire, the subjective sensation estimated in the Salanova questionnaire shows the older and with more years of experience teachers as those with the greatest symptoms of techno-anxiety, techno-fatigue, techno-skepticism, and techno-inefficacy (see Table 8).

**TABLE 8:** Pearson correlations between Salanova (2003) technostress questionnaire and age and years of experience.

	**Age**	**Years of experience**	**Total**	**Techno-fatigue**	**Techno-anxiety**	**Techno-inefficacy**	**Techno-skepticism**
Age	1						
Years of experience	0.672**	1					
Total	0.138*	0.204**	1				
Techno-fatigue	0.113	0.150*	0.857**	1			
Techno-anxiety	0.146*	0.181**	0.895**	0.744**	1		
Techno-inefficacy	0.268**	0.259**	0.806**	0.572**	0.760**	1	
Techno-skepticism	0.254**	0.298**	0.757**	0.568**	0.603**	0.646**	1

*p < 0.05; **p < 0.01.

Regarding job performance, statistically significant differences were observed, as in the previous questionnaires, depending on the type of university considered. This indicates that online university teachers were more likely to view technology as a tool to help them in their performance [3.8578 vs. 3.1715; *t*(237) = 4.615, *p* < 0.001; *d* = 0.68], compared to the face-to-face university teachers.

Similarly, older teachers (*r* = −0.163; *p* < 0.05) and those with more years of experience (*r* = −0.172; *p* < 0.05) felt that technology did not help them in their teaching role; thus, there were no differences in job performance when taking gender into account.

### Correlation Analysis

The joint influence of both technostress measures (objective/subjective) was corroborated with the results obtained in the correlations between both instruments. In result, a joint influence of the objective and subjective measures of technostress was observed.

Wang and Li’s (2019) objective technostress questionnaire and Salanova’s (2003) subjective measure of technostress presented a statistically significant positive correlation (*r* = 0.703; *p* < 0.001) for the study sample. Taking into account the subfactors that comprise both scales, the misfit of teachers’ abilities to the technological demands of the universities (ADT) provoked greater subjective sensations of technostress and, in particular, techno-fatigue (*r* = 0.625; *p* < 0.001), techno-anxiety (*r* = 0.620; *p* < 0.001), and techno-inefficacy (*r* = 0.668; *p* < 0.001) (see Table 9).

**TABLE 9:** Pearson correlations between the technostress scales and subscales of the instruments used.

	Salanova, 2003	Wang and Li, 2019	**SKE**	**FAT**	**ANS**	**INEF**	**ADO**	**NSO**	**ADT**	**NST**	**PPF**
Salanova, 2003	1										
Wang and Li, 2019	0.703**	1									
ESC	0.757**	0.638**	1								
FAT	0.857**	0.604**	0.568**	1							
ANS	0.895**	0.629**	0.603**	0.744**	1						
INEF	0.806**	0.682**	0.646**	0.572**	0.760**	1					
ADO	0.610**	0.848**	0.544**	0.550**	0.571**	0.643**	1				
NSO	0.518**	0.844**	0.464**	0.438**	0.453**	0.469**	0.570**	1			
ADT	0.695**	0.904**	0.585**	0.625**	0.620**	0.668**	0.826**	0.677**	1		
NST	0.666**	0.895**	0.644**	0.548**	0.579**	0.639**	0.702**	0.732**	0.755**	1	
PPF	0.537**	0.817**	0.514**	0.437**	0.484**	0.517**	0.549**	0.666**	0.619**	0.688**	1

**p < 0.01.

### Proposed Technostress Models

The above significant correlations indicate a differential pattern in the type of technostress as well as how technostress, depending on the university, gender, age, and teaching experience, affects job performance during the period of confinement.

To examine in detail the relative weight of each of the variables considered, a SEM was used, where gender, age, years of experience, professional category, and the objective/subjective measures of technostress were considered as predictor variables of job performance.

The results show that the objective (β = −34) and subjective (β = −16) measures of technostress, as well as the teaching format of the university (online or face-to-face) (β = −16), served as explanatory variables of job performance. Given the provisional results obtained, two predictive models of job performance had been replicated, differentiating by the type of teaching methods (online or face-to-face).

Both models obtained goodness-of-fit indices which were considered good and supported the theoretical models used for this study (see Table 10).

**TABLE 10:** Goodness of fit indices of the proposed models.

	**Online**	**Face-to-face**
RMR	0.068	0.074
AGFI	0.989	0.988
GFI	0.993	0.993
NFI	0.990	0.989
RFI	0.986	0.985

**FIGURE 1:**
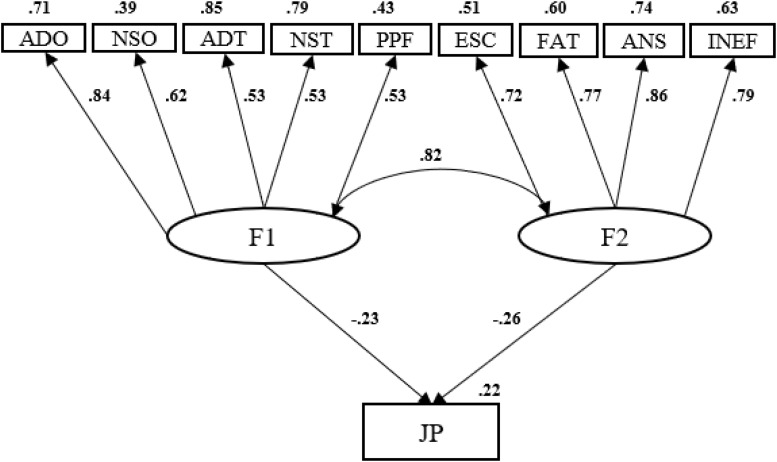
Predictive model of the performance of face-to-face university teachers based on technostress.

For face-to-face university teachers, 22% of the explained variance in work performance could be explained through the technostress measures considered, with the same contribution for the objective variables (β = −23), rather than subjective (β = −26) variables of technostress (see Figure 1).

**FIGURE 2:**
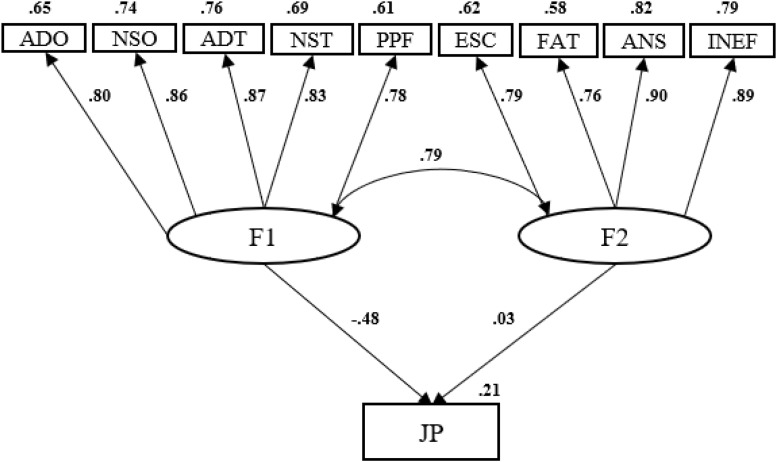
Predictive model of the performance of online university teachers based on technostress.

For online teachers, almost the same amount of variability in work performance was obtained as for teachers in the face-to-face modality (21%); in this case, the predictive weight focused exclusively on the objective measures of technostress was examined using the Wang and Li (2019) questionnaire (β = −48) (see Figure 2).

The very diverse context in which teaching functions are carried out by professors from both face-to-face and online universities makes it necessary to corroborate whether the significant correlations remain between the subfactors, or if, on the contrary, there are variations that allow technostress to be characterized differently in both contexts.

For the general scales, the significant correlations between the objective technostress scale (Wang and Li, 2019) and the subjective technostress scale (Salanova, 2003) were maintained, yet, a greater intensity was shown for online university teachers (*r* = 0.727; *p* < 0.001) compared to face-to-face university teachers (*r* = 0.674; *p* < 0.001).

By analyzing the subscales of both instruments, there appeared to be differences in the sensation of technostress. In fact, teachers from the online universities showed a greater sense of techno-inefficacy when the demands of the organization exceeded the skills of its workers (ADO; *r* = 0.668; *p* < 0.001), when the university did not provide the necessary resources for teachers to carry out their functions (NSO; *r* = 0.653; *p* < 0.001), and when teachers did not have the necessary skills for the established technological demands (ADT; *r* = 0.728; *p* < 0.001) (see Table 11).

**TABLE 11:** Pearson correlations between the technostress scales and subscales of the instruments used depending on the university: online (below) face-to-face (above).

	Salanova, 2003	Wang and Li, 2019	**SKE**	**FAT**	**ANS**	**INEF**	**ADO**	**NSO**	**ADT**	**NST**	**PF**
Salanova, 2003	1	0.674**	0.729**	0.857**	0.893**	0.772**	0.587**	0.431**	0.678**	0.647**	0.469**
Wang and Li, 2019	0.727**	1	0.605**	0.593**	0.603**	0.635**	0.842**	0.793**	0.883**	0.886**	0.779**
ESC	0.792**	0.607**	1	0.515**	0.563**	0.603**	0.520**	0.369**	0.549**	0.636**	0.462**
FAT	0.839**	0.563**	0.658**	1	0.743**	0.534**	0.557**	0.379**	0.638**	0.523**	0.372**
ANS	0.888**	0.666**	0.666**	0.713**	1	0.723**	0.539**	0.370**	0.612**	0.568**	0.425**
INEF	0.863**	0.731**	0.691**	0.613**	0.834**	1	0.607**	0.349**	0.616**	0.618**	0.462**
ADO	0.597**	0.860**	0.504**	0.446**	0.595**	0.668**	1	0.497**	0.799**	0.699**	0.511**
NSO	0.633**	0.885**	0.558**	0.481**	0.595**	0.653**	0.657**	1	0.603**	0.672**	0.567**
ADT	0.674**	0.911**	0.544**	0.500**	0.588**	0.728**	0.881**	0.699**	1	0.717**	0.549**
NST	0.641**	0.869**	0.537**	0.522**	0.539**	0.587**	0.627**	0.768**	0.745**	1	0.641**
PPF	0.602**	0.798**	0.487**	0.491**	0.559**	0.514**	0.510**	0.725**	0.585**	0.648**	1

**p < 0.01.

In the case of face-to-face university teachers, more varied symptoms of technostress were observed depending on technological skills, organizational skills, and resources. In this aspect, teachers had a greater sense of techno-inefficacy when the demands of the institution exceeded the skills of their workers (ADO; *r* = 0.607; *p* < 0.001), when the university did not have the necessary technological resources (NST; *r* = 0.616; *p* < 0.001), or when teachers did not have the necessary skills for the established technological demands (ADT; *r* = 0.618; *p* < 0.001). The lack of technological ability of face-to-face teachers produced a feeling of techno-anxiety (*r* = 0.612; *p* < 0.001), techno-fatigue (*r* = 0.638; *p* < 0.001) and techno-inefficacy. This technological inability led to greater discomfort or subjective sensation of technostress among face-to-face teachers.

Lastly, it should be noted that when the university did not have the necessary technological resources to exercise teaching functions, higher scores for techno-skepticism (*r* = 0.636; *p* < 0.001) were observed among face-to-face teachers (see Table 11).

## Discussion

This research represents an advancement in the study of technostress for university teaching staff. A multidimensional analysis of the phenomenon was carried out in which personal variables (i.e., variables associated with objective and subjective technostress) and job performance were analyzed. In addition, it verified that the person-environment fit theory (P-E fit theory) developed by Wang and Li (2019) is maintained in the Spanish population.

This is the first study of technostress in university teaching staff to be carried out at a time of home confinement, when, for the first time, university education was forced to develop completely online, regardless of direct teacher-student contact.

The improvisation to which both teachers and students had to resort, subjected both groups to a level of stress that is not recommended for an adequate level of academic performance, as evidenced in this study and recent research on this topic (Li and Wang, 2020; Özgür, 2020; Penado Abilleira et al., 2020; Schildkamp et al., 2020; Wang et al., 2020). Hence, this study is interested in understanding which factors affect this situation the most. It has become clear how Spanish university teachers presented different levels of technostress when carrying out their teaching tasks during the period of confinement caused by the COVID-19 pandemic in Spain.

It was shown that older teachers and those with more years of experience (consequently holding the highest professional job categories), are those who have suffered negative consequences of technology to a greater extent during the confinement period. Contrary to what has been pointed out in other studies (Li and Wang, 2020), there appears to be an influence of gender on the level of technostress observed in university teachers, with women suffering more negative effects of technology compared to men. The influence of gender on technostress is consistent with other studies on student populations (Wang et al., 2020).

The weights of the objective variables or subjective feeling of technostress differed considerably between teachers based on the type of university (i.e., online or face-to-face). Nonetheless, the impact on job performance due to technostress was the same, with percentages of variability found to be close to identical.

Professors at online teaching universities pointed out objective aspects or difficulties in the technological resources provided by the organization in which they worked as the main difficulty in their work performance during the period of confinement caused by the COVID-19 pandemic. In this sense, the variables described in the Wang and Li (2019) questionnaire fully explain the performance problems of online teachers, with a total absence in the weight of variables or subjective feelings associated with technology.

In contrast, teachers from face-to-face universities had to make the greatest adaptations during confinement by incorporating technological platforms and resources that were formerly used less in their previous teaching environment.

The findings of this study show that the response of Spanish universities to the COVID-19 pandemic generated a lack of confidence in technology for online university teachers as well as a need to improve job performance. This decrease in job performance could be explained by objective aspects such as a lack of resources or instructions from their organization in order to carry out the new functions that they were expected to fulfill during the period of confinement. In the case of the face-to-face university teachers, a combined effect of objective aspects (e.g., lack of skills to comply with the instructions given by their university during the shift to online teaching or an absence of instructions), together with subjective feelings of techno-inefficacy, was observed.

The results obtained contrast with what has been established thus far by other authors which indicate that the employees of organizations with an environment characterized by high centralization and high innovation are the more likely to report a feeling of technostress (*technostrain*) in comparison with employees of less centralized and more innovative organizations (Wang et al., 2008). Based on these conclusions, it would be possible to expect that online teachers experience the effect of more subjective components of technostress, yet, the results obtained demonstrate the opposite.

It is necessary to incorporate measures that reduce stress associated with the use of technology in higher education. More specifically, these measures should be aimed at avoiding the consequences of technostress at an organizational level, such as absenteeism and reduced performance of technology users, especially due to the nonuse or misuse of technology in the workplace (Tu et al., 2005).

Educational authorities should aim to avoid an increase in workload due to the pressure to work faster or to work within more stringent schedules (Tarafdar et al., 2015). Additionally, educational authorities should aim to facilitate the education and training for face-to-face teachers in the use of various technologies (Graves and Karabayeva, 2020).

Due to the subjective feeling of technostress that teachers from face-to-face universities claim to suffer in comparison with their peers at online universities, various measures must be incorporated into face-to-face universities. Examples include actions that favor literacy facilitation, integration of technological and pedagogical knowledge until reaching the “knowledge of technological pedagogical content” (TPACK), and continuous teacher professional development (TPD) (Rienties et al., 2013; Özgür, 2020; Schildkamp et al., 2020). Technical support provision and involvement facilitation must also be added as strategies as these strategies have already been shown to inhibit technostress in university teachers in other populations (Li and Wang, 2020).

In the case of online teachers with experience in using technology, an intervention should be focused on informing or guiding them toward choosing the correct technology to carry out the entrusted task (Wang and Haggerty, 2011).

In both online and face-to-face institutions, training, rehearsal, a series of courses with special reference to less-skilled employees, teamwork, and sharing knowledge should always be available to employees to improve job performance and reduce the negative effect of techno-complexity (Al-Ansari and Alshare, 2019).

Finally, possible limitations of the obtained results should be pointed out. The most important limitation is related to the study sample, such that it would be convenient to expand the sample size in subsequent studies, as well as the length of data collection. The exceptionality of the situation experienced during confinement may have raised technostress. Therefore, it will be necessary to contrast these results with data obtained when the state of exceptionality generated by the COVID-19 pandemic disappears, and thus confirm the stability thereof.

It will also be necessary to triangulate the information through other analysis methodologies that provide a qualitative view of the phenomenon, using techniques such as interviews, observation, and discussion groups in order to allow a deeper understanding of the phenomenon. Likewise, it will be necessary to investigate more about the technostress associated with the different types of higher education that derive from the use of ICT (e.g., online teaching, blended learning, face-to-face teaching, e-learning, b-learning, teachings to distance, etc.), to further refine the stress-generating factors that can affect job performance for each type.

New forms of teaching and learning have been developed in recent years, and the circumstances arising from the current pandemic is accelerating the process. The social distancing regulations that have been imposed make it clear that face-to-face learning must adapt in order to remain competitive within the “post-pandemic reality.” It is important to face the technological transition in the context of higher education with confidence while attempting to avoiding risks derived from the shift toward digitization.

## Data Availability Statement

The raw data supporting the conclusions of this article will be made available by the authors, without undue reservation.

## Ethics Statement

Ethical review and approval was not required for the study on human participants in accordance with the local legislation and institutional requirements. The patients/participants provided their written informed consent to participate in this study.

## Author Contributions

All authors listed have made a substantial, direct and intellectual contribution to the work, and approved it for publication.

## Conflict of Interest

The authors declare that the research was conducted in the absence of any commercial or financial relationships that could be construed as a potential conflict of interest.
